# Genetic Cross-Interaction between *APOE* and *PRNP* in Sporadic Alzheimer's and Creutzfeldt-Jakob Diseases

**DOI:** 10.1371/journal.pone.0022090

**Published:** 2011-07-20

**Authors:** Olga Calero, María J. Bullido, Jordi Clarimón, Ana Frank-García, Pablo Martínez-Martín, Alberto Lleó, María Jesús Rey, Alberto Rábano, Rafael Blesa, Teresa Gómez-Isla, Fernando Valdivieso, Jesús de Pedro-Cuesta, Isidro Ferrer, Miguel Calero

**Affiliations:** 1 Centro de Investigación Biomédica en Red sobre Enfermedades Neurodegenerativas (CIBERNED), Madrid, Spain; 2 Unidad de Encefalopatías Espongiformes, Centro Nacional de Microbiología, Instituto de Salud Carlos III (CNM-ISCIII), Madrid, Spain; 3 Centro de Biología Molecular Severo Ochoa (CSIC-UAM), Madrid, Spain; 4 Institute of Sanitary Research “Hospital la Paz” (IdIPaz), Madrid, Spain; 5 Neurology Department, Hospital de la Santa Creu i Sant Pau, Universitat Autònoma de Barcelona, Barcelona, Spain; 6 Neurology Service, Hospital Universitario La Paz (UAM), Madrid, Spain; 7 Alzheimer Disease Research Unit, CIEN Foundation, Carlos III Institute of Health, Alzheimer Center Reina Sofia Foundation, Madrid, Spain; 8 Banco de Tejidos Neurológicos Universidad de Barcelona-Hospital Clínico, Barcelona, Spain; 9 Banco de Tejidos de la Fundación CIEN, CIEN Foundation, Carlos III Institute of Health, Alzheimer Center Reina Sofia Foundation, Madrid, Spain; 10 Área de Epidemiologia Aplicada, Centro Nacional de Epidemiología, Instituto de Salud Carlos III (CNM-ISCIII), Madrid, Spain; 11 Institute of Neuropathology (INP), IDIBELL-Hospital Universitari de Bellvitge, Faculty of Medicine, University of Barcelona, 08907 Hospitalet de LLobregat, Barcelona, Spain; University of Melbourne, Australia

## Abstract

Alzheimer's disease (AD) and Creutzfeldt-Jakob disease (CJD) represent two distinct clinical entities belonging to a wider group, generically named as conformational disorders that share common pathophysiologic mechanisms. It is well-established that the *APOE* ε4 allele and homozygosity at polymorphic codon 129 in the *PRNP* gene are the major genetic risk factors for AD and human prion diseases, respectively. However, the roles of *PRNP* in AD, and *APOE* in CJD are controversial. In this work, we investigated for the first time, *APOE* and *PRNP* genotypes simultaneously in 474 AD and 175 sporadic CJD (sCJD) patients compared to a common control population of 335 subjects. Differences in genotype distribution between patients and control subjects were studied by logistic regression analysis using age and gender as covariates. The effect size of risk association and synergy factors were calculated using the logistic odds ratio estimates. Our data confirmed that the presence of *APOE* ε4 allele is associated with a higher risk of developing AD, while homozygosity at *PRNP* gene constitutes a risk for sCJD. Opposite, we found no association for *PRNP* with AD, nor for *APOE* with sCJD. Interestingly, when AD and sCJD patients were stratified according to their respective main risk genes (*APOE* for AD, and *PRNP* for sCJD), we found statistically significant associations for the other gene in those strata at higher previous risk. Synergy factor analysis showed a synergistic age-dependent interaction between *APOE* and *PRNP* in both AD (SF = 3.59, p = 0.027), and sCJD (SF = 7.26, p = 0.005). We propose that this statistical epistasis can partially explain divergent data from different association studies. Moreover, these results suggest that the genetic interaction between *APOE* and *PRNP* may have a biological correlate that is indicative of shared neurodegenerative pathways involved in AD and sCJD.

## Introduction

Both Alzheimer's disease (AD) and Creutzfeldt-Jakob disease (CJD) are brain amyloidoses associated with old age. Although, they represent two distinct clinical entities, AD and CJD are now considered as part of a wider group generically named as conformational disorders, and more specifically brain amyloidosis. These two disorders share common pathophysiologic mechanisms involving protein deposits in the brain due to the conversion of a soluble, normal protein into an insoluble, aggregated form leading to fatal degeneration [1]–[5]. From an etiological standpoint, both AD and CJD can be classified as sporadic and familial forms (and also as acquired in CJD). The sporadic cases (sporadic CJD –sCJD- and late onset AD) represent the most frequent forms; and although of unknown aetiology, several genetic risk factors have been described. The *APOE* ε4 allele is a major risk factor for AD [6], [7]; whereas homozygosity at polymorphic codon 129 (Met to Val) in the *PRNP* gene is a well-established susceptibility marker for human prion diseases [8]. However, the role of *APOE* in CJD and *PRNP* in AD is matter of discussion.

Previous studies on the distribution of *PRNP* codon 129 in AD, or the *APOE* ε4 allele in CJD yielded conflicting results. Some studies found an association between the *PRNP* codon 129 and AD [9]–[11], while others did not [12]–[19]. Likewise, some studies found an association between the presence of the *APOE* ε4 allele and CJD [20], [21], while others reports were negative [22]–[27].

Since these disorders share several features, a genetic interaction between these genes might determine susceptibility to both diseases or modulate the clinical phenotypes. A potential genetic interaction between *APOE* and *PRNP* genes has been rarely studied for CJD or AD. Furthermore, to our knowledge, an analysis of the potential epistasis of these genes in both disorders by using the same control population and statistical approach has never been materialized. In this study, we investigated a possible genetic interaction between *APOE* ε4 allele and the polymorphic codon 129 of the *PRNP* gene in both AD and sCJD populations.

## Results

In this study, two different patient (AD and sCJD) and one control populations were analyzed. The control group consisted of 335 subjects; 198 (59.1%) women and 137 (40.9%) men. Age at study inclusion for control individual followed a non-normal distribution with a mean of 73.2 years and standard deviation of 11.0 years. The AD group consisted of 474 patients diagnosed with probable AD according to the NINCDS-ADRDA criteria; 323 were women (68.1%) and 151 were men (31.9%). Age at onset for control individuals followed a non-normal distribution with a mean of 74.5±8.2 years. The sCJD group (n = 175) included 140 (80.0%) definite, neuropathologically verified patients, and 35 (20.0%) probable cases that were diagnosed with sCJD. The gender distribution was 87 (49.7%) women and 88 (50.3%) men. Age at onset for this group followed a non-normal distribution with a mean of 67.3±11 years. No statistically significant differences in gender distribution or age at onset were found between definite and probable sCJD cases.

Analysis of gender distribution showed statistically significant differences between AD (p = 0.010) or sCJD (p = 0.049) patients and the control group. Analysis of age distribution yielded statistically significant differences for sCJD patients (p<0.001), and for AD patients (p = 0.006) compared to the control group. Mean age and gender distribution of the control population were intermediate between those of AD and sCJD cases, supporting its validity as a common control group. In order to correct for potential effects of the differential gender and age distributions, odds ratios and synergy factors were obtained from logistic regression algorithms controlling by age and gender.

The distribution of the polymorphic codon 129 of *PRNP* and *APOE* genotypes in control subjects, AD and sCJD patients are shown in Table 1. *APOE* polymorphic variants at codon 112 and codon 158, as well as the allelic haplotypes ε2/ε3/ε4, were in Hardy-Weinberg equilibrium in the three populations studied. As expected, the polymorphic codon 129 of the *PRNP* gene was in Hardy-Weinberg equilibrium in the healthy control and AD populations, but in clear disequilibrium (p<0.0001) in the sCJD population.

**Table 1:** *APOE* and *PRNP* codon 129 genotypic and allelic frequencies in control subjects (Cont) and patients (AD, sCJD).

	*PRNP* codon 129		*APOE* Genotypic frequency, n(%)						*APOE* ε4 status^a^, n(%)	
		n	ε2/ε2	ε2/ε3	ε2/ε4	ε3/ε3	ε3/ε4	ε4/ε4	ε4−	ε4+
**Cont**	**Genotypes**									
	M129M	129(100.0)	0(0.0)	14(10.9)	0(0.0)	101(78.3)	13(10.1)	1(0.8)	115(89.1)	14(10.9)
	M129V	165(100.0)	1(0.6)	15(9.1)	0(0.0)	122(73.9)	27(16.4)	0(0.0)	138(83.6)	27(16.4)
	V129V	41(100.0)	0(0.0)	10(24.4)	0(0.0)	22(53.7)	8(19.5)	1(2.4)	32(78.0)	9(22.0)
	Total	335(100.0)	1(0.3)	39(11.6)	0(0.0)	245(73.1)	48(14.3)	2(0.6)	285(85.1)	50(14.9)
	**Alleles**									
	M129	423(100.0)	1(0.2)	43(10.2)	0(0.0)	324(76.6)	53(12.5)	2(0.5)	368(87.0)	55(13.0)
	V129	247(100.0)	1(0.4)	35(14.2)	0(0.0)	166(6.5)	43(17.4)	2(0.8)	202(81.8)	45(18.2)
	Total	670(100.0)	2(0.3)	78(11.6)	0(0.0)	490(73.1)	96(14.3)	4(0.6)	570(85.1)	100(14.9)
**AD**	**Genotypes**									
	M129M	215(100.0)	1(0.5)	8(3.7)	6(2.8)	106(49.3)	80(37.2)	14(6.5)	115(53.5)	100(46.5)
	M129V	212(100.0)	0(0.0)	9(4.2)	3(1.4)	117(55.2)	71(33.5)	12(5.7)	126(59.4)	86(40.6)
	V129V	47(100.0)	0(0.0)	4(8.5)	0(0.0)	26(55.3)	12(25.5)	5(10.6)	30(63.8)	17(36.2)
	Total	474(100.0)	1(0.2)	21(4.4)	9(1.9)	249(52.5)	163(34.4)	31(6.5)	271(57.2)	203(42.8)
	**Alleles**									
	M129	642(100.0)	2(0.3)	25(3.9)	15(2.3)	329(51.2)	231(36.0)	40(6.2)	356(55.5)	286(44.5)
	V129	306(100.0)	0(0.0)	17(5.6)	3(1.0)	169(55.2)	95(31.0)	22(7.2)	186(60.8)	120(39.2)
	Total	948(100.0)	2(0.2)	42(4.4)	18(1.9)	498(52.5)	326(34.4)	62(6.5)	542(57.2)	406(42.8)
**sCJD**	**Genotypes**									
	M129M	112(100.0)	0(0.0)	12(10.7)	1(0.9)	75(67.0)	24(21.4)	0(0.0)	87(77.7)	25(22.3)
	M129V	36(100.0)	0(0.0)	3(8.3)	0(0.0)	30(83.3)	1(2.8)	2(5.6)	33(91.7)	3(8.3)
	V129V	27(100.0)	0(0.0)	5(18.5)	0(0.0)	13(48.1)	9(33.3)	0(0.0)	18(66.7)	9(33.3)
	Total	175(100.0)	0(0.0)	20(11.4)	1(0.6)	118(67.4)	34(19.4)	2(1.1)	138(78.9)	37(21.1)
	**Alleles**									
	M129	260(100.0)	0(0.0)	27(10.4)	2(0.8)	180(69.2)	49(18.8)	2(0.8)	207(79.6)	53(20.4)
	V129	90(100.0)	0(0.0)	13(14.4)	0(0.0)	56(62.2)	19(21.1)	2(2.2)	69(76.7)	21(23.3)
	Total	350(100.0)	0(0.0)	40(11.4)	2(0.6)	236(67.4)	68(19.4)	4(1.1)	276(78.9)	74(21.1)

a *APOE* ε4 status: ε4− = no copies of ε4 allele, ε4+ = one or two copies of ε4 allele.

### *APOE* and *PRNP* in AD

As shown in Table 2, and previously repeatedly described in the literature, we found an increased risk of AD in *APOE* ε4 allele carriers (OR = 4.51). Interestingly, when this association was studied in different strata according to *PRNP* codon 129 genotypes, we observed an increase in the OR associated with the presence of M129 alleles. Thus, the highest OR (7.28) was observed in M129M homozygous, while the lowest OR in V129V homozygous (2.06). The Mantel-Haenszel test for homogeneity indicated that these two homozygous strata were statistically significant different (p = 0.026). The fact that the OR for each stratum is different and the global OR appears to be an average OR suggested an interaction between *APOE* and *PRNP*, rather than *PRNP* acting as a confounding factor.

**Table 2:** Odds ratios for the association between Alzheimer's disease and *APOE* ε4 carriers (vs. *APOE* ε4 non-carriers) among different strata defined by *PRNP* codon 129 genotypes.

Subjects	OR (95% CI)	p-value
All subjects	4.51 (3.16–6.45)	<0.0001
M129M	7.28 (3.92–13.50)	<0.0001
M129V	3.92 (2.36–6.50)	<0.0001
V129V	2.06 (0.77–5.48)	0.15

Additionally, we also observed a decreased risk of AD in *APOE* ε2 allele carriers (OR = 0.50, 95% CI: 0.30–0.82, p = 0.006). Similar to the results with the *APOE* ε4 allele carriers, when this analysis was performed in the 3 different strata defined by *PRNP* codon 129, we found a tendency to higher protection associated with the presence of *PRNP* V129 alleles (M129M: OR = 0.61, 95% CI: 0.28–1.31, p = 0.20; and M129V: OR = 0.55, 95% CI: 0.25–1.21, p = 0.14; and V129V: OR = 0.25, 95% CI: 0.07–0.91, p = 0.036).

The analysis of AD risk related to the status of *PRNP* codon 129 (Table 3) indicated a non-significant association trend for the M129 allele (OR = 1.23, p = 0.057), as well as for the M129M (OR = 1.47) and M129V (OR = 1.16) genotypes compared to homozygous V129V for the whole AD population. However, after stratification of AD subjects according to their *APOE* ε4 status, we found a clear association (OR = 3.94, p = 0.007) for M129M homozygous only in *APOE* ε4 carriers. In this *APOE* ε4+ stratum, the analysis of dose effect indicated a two-fold increase in AD risk (OR = 2.02, p = 0.004) for each *PRNP* M129 allele carried. A similar analysis for *APOE* ε2 carriers did not show any significant association in any strata.

**Table 3:** Odds ratios for the association between Alzheimer's disease and *PRNP* codon 129 in different strata defined by *APOE* ε4 allele status (V129V genotype and V129 allele are taken as reference or non-exposed).

Subjects	OR (95% CI)	p-value
**All subjects**		
**Genotypes**		
M129M	1.47 (0.91–2.36)	0.11
M129V	1.16 (0.72–1.85)	0.54
**Alleles**		
M129	1.23 (0.99–1.52)	0.057
***APOE*** ** ε4− subjects** a		
**Genotypes**		
M129M	1.07 (0.61–1.90)	0.81
M129V	1.00 (0.57–1.74)	0.99
**Alleles**		
M129	1.05 (0.81–1.36)	0.71
***APOE*** ** ε4+ subjects** b		
**Genotypes**		
M129M	3.94 (1.46–10.6)	0.007
M129V	1.83 (0.72–4.63)	0.21
**Alleles**		
M129	2.02 (1.26–3.24)	0.004

a *APOE* ε4− = no copies of ε4 allele;

b *APOE* ε4+ = one or two copies of ε4 allele.

Altogether, these findings suggested that the M129 allele of the *PRNP* gene is a susceptibility genetic risk factor of AD only in *APOE* ε4 carriers and prompted us to further explore the potential genetic interaction between *APOE* and *PRNP* in the AD population. In order to study the combined gene effects, we analyzed the risk of developing AD for subjects carrying both traits (*APOE* ε4 carriers and *PRNP* M129M homozygous) compared to subjects without these traits (Table 4). M129M homozygous subjects carrying at least one *APOE* ε4 allele have a 7.68-fold (p<0.0001) increased risk of developing AD compared to subjects V129V with no *APOE* ε4 alleles, which is higher than the expected combined effect of the individual contributions of *APOE* ε4 and *PRNP* M129M, suggesting gene-gene interaction or epistasis (Table 4, upper).

**Table 4:** Synergy in AD population between *APOE* and *PRNP* genes.

	*PRNP* codon 129	*APOE* ε4 allele^a^	Controls	AD	OR (95% CI)	SF (p-value)
**M129M vs. V129V** b	−	−	32	30	Reference	
	+	−	115	115	1.07 (0.61–1.87)	
	−	+	9	17	2.00 (0.77–5.21)	
	+	+	14	100	7.68 (3.66–16.55)	3.59 (0.027)
**M129 vs. V129** c	−	−	202	186	Reference	
	+	−	368	356	1.05 (0.82–1.35)	
	−	+	45	120	2.90 (1.92–4.31)	
	+	+	55	286	5.65 (3.98–8.02)	1.86 (0.018)

a *APOE* ε4 allele: − = no copies of ε4 allele, + = one or two copies of ε4 allele;

b OR calculated by a logistic regression model controlling by age, linear values, and gender and using the *PRNP*×*APOE* interaction factor as third independent variable;

c OR calculated by chi-square.

In order to measure the size and significance of the interaction, we performed a synergy factor (SF) analysis following reported methods. According to this analysis, we obtained a SF of 3.59, which is statistically significant different (p = 0.027) from 1; indicating a synergistic interaction between two risk factors, *APOE* ε4 and M129M (Table 4, upper). A similar analysis by *PRNP* alleles (M129 vs. V129) indicated a SF of 1.86 (p = 0.018) for the interaction *APOE* ε4 and a M129 allele (Table 4, bottom).

### *PRNP* and *APOE* in sCJD

As shown in Table 5, an increased risk for sCJD was associated with M129M (OR = 4.61) and V129V (OR = 3.20) homozygous compared to M129V heterozygous at codon 129 of the *PRNP* gene. Interestingly, when these associations were studied in different strata according to the *APOE* ε4 status, we observed an important increase in the OR for *APOE* ε4 carriers compared to non-ε4 carriers (28.5 vs. 3.5 for M129M and 13.2 vs. 2.43 for V129V). Mantel-Haenszel tests for homogeneity indicated that the *APOE* ε4 carriers were statistically significant different from *APOE* ε4 non-carriers for the M129M comparison (p = 0.022). These data, as in the case of the AD population, suggested an interaction between *APOE* and *PRNP* in sCJD. The analysis of the *APOE* ε4 allele dose effect (no, one or two copies of *APOE* ε4 allele) showed similar results, although of lower magnitude to the ones observed for the ε4+ and no-ε4 allele comparison (M129M OR = 2.26 p = 0.023; M129V OR = 0.68 p = 0.48; V129V OR = 1.26 p = 0.68; Homozygosity OR = 1.89 p = 0.032), suggesting that the effect of *APOE* ε4 allele was dose dependent.

**Table 5:** Odds ratios for the association between CJD and *PRNP* codon 129 in different strata defined by *APOE* ε4 allele status (M129V genotype is taken as reference or non-exposed).

Subjects	OR (95% CI)	p-value
**All subjects**		
M129M	4.61 (2.89–7.35)	<0.0001
V129V	3.20 (1.69–6.05)	0.0004
***APOE*** ** ε4− subjects** a		
M129M	3.50 (2.13–5.74)	<0.0001
V129V	2.43 1.18–5.03	0.017
***APOE*** ** ε4+ subjects** b		
M129M	28.5 (5.63–144.3)	<0.0001
V129V	13.2 (2.25–76.9)	0.004

a *APOE* ε4− = no copies of ε4 allele;

b *APOE* ε4+ = one or two copies of ε4 allele.

We found no association between *APOE* ε4 allele status and sCJD for the total population (OR = 1.46, p = 0.13) (Table 6). However, when the different genotypes of *PRNP* codon 129 were analyzed individually, a statistical significant risk associated with the *APOE* ε4 allele was observed in the M129M homozygous stratum (OR = 2.47, p = 0.017). We also found a similar trend, although not statistically significant, in the V129V homozygous stratum. Opposite, a non significant protector effect for the *APOE* ε4 allele was found in M129V heterozygous stratum. Mantel-Haenszel test indicated significant differences between the M129M homozygous and M129V heterozygous strata (p = 0.022) or homozygous (M129M and V129V) vs. M129V heterozygous (p = 0.027). We did not find association for the *APOE* ε2, neither in the unselected domain (all sCJD/control populations), nor in any of the subpopulations defined by *PRNP* codon 129 genotypes.

**Table 6:** Odds ratios for the association between CJD and *APOE* ε4 carriers (vs. *APOE* ε4 non-carriers) among different strata defined by *PRNP* codon 129 genotypes.

Subjects	OR (95% CI)	p-value
All subjects	1.46 (0.89–2.38)	0.13
M129M	2.47 (1.18–5.16)	0.017
M129V	0.30 (0.067–1.34)	0.12
V129V	1.55 (0.46–5.25)	0.48
Homozygous (M129M+V129V)	2.14 (1.16–3.95)	0.015

In order to study the combined gene effects between *APOE* and *PRNP* in sCJD, we analyzed the risk of developing sCJD for subjects carrying both traits (*APOE* ε4 carriers and *PRNP* homozygous) compared to subjects without these traits (Table 7 top). *PRNP* homozygous subjects carrying at least one *APOE* ε4 allele have a 6.83-fold (p<0.0001) increased risk of developing sCJD than subjects M129V heterozygous with no *APOE* ε4 alleles. This increased risk appears to be higher than a multiplicative effect of each independent factor, suggesting gene-gene interaction or epistasis. Synergy factor analysis yielded a SF of 7.26, which is statistically significant different (p = 0.005) from 1; indicating a synergistic interaction between two risk factors, *APOE* ε4 and *PRNP* homozygous. A similar analysis by *PRNP* genotypes (M129M vs. M129V) indicated a SF of 8.48 (p = 0.003) for the interaction *APOE* ε4 and *PRNP* M129M genotype (Table 7, bottom).

**Table 7:** Synergy in sCJD population between *APOE* and *PRNP* genes.

	*PRNP* codon 129	*APOE* ε4 allele^a^	Controls	sCJD	OR (95% CI)	SF (p-value)
**Homozygous vs. Heterozygous** b	−	−	138	33	Reference	
	+	−	147	105	3.25 (2.01–5.23)	
	−	+	27	3	0.29 (0.07–1.30)	
	+	+	23	34	6.83 (3.45–13.51)	7.26 (0.005)
**M129M vs. M129V** b	−	−	138	35	Reference	
	+	−	115	90	3.45 (2.11–5.65)	
	−	+	27	4	0.29 (0.07–1.30)	
	+	+	14	27	8.48 (3.87–18.61)	8.48 (0.003)

a *APOE* ε4 allele: − = no copies of ε4 allele, + = one or two copies of ε4 allele;

b OR calculated by a logistic regression model controlling by age as a linear variable and gender, and introducing the *PRNP*×*APOE* interaction factor as third independent variable.

### *APOE* and *PRNP* influence on disease onset and duration

We further explored the influence of these genetic factors on disease onset for both AD and sCJD populations. In AD, we observed that *APOE* ε4 carriers had an earlier onset than non-ε4 carriers (72.8±7.0 years vs. 75.7±8.8 years, p<0.001), in agreement with previous reports [6], [28]. We also observed a non-significant trend for later onset of *APOE* ε2 carriers compared to non-ε2 carriers (76.8±10.8 years vs. 74.3±8.0 years, p<0.50), as previously reported [29]. On the other hand, we did not find any association with onset for the 3 different genotypes related to the codon 129 of *PRNP* (M129M: 74.1±8.1 years, M129V: 74.7±8.0 years, V129V: 75.5±9.5 years, p = 0.62).

For sCJD, we found a non-significant tendency for earlier onset associated with the *PRNP* V129 allele (M129M: 68.4±10.6 years, M129V 66.6±11.3 years, V129V: 63.0±11.6 years, p = 0.13). Regarding *APOE*, we observed a later onset for *APOE* ε2 carriers compared to non-ε2 carriers (72.2±7.8 years vs. 66.7±11.2 years, p = 0.046) in agreement to previous reports [30]; while we failed to detect any effect for the *APOE* ε4 allele (67.8±10.5 years vs. 67.2±11.2 years, p = 0.75), also in agreement with previous results [27].

Additionally, we also explored the influence of these genetic factors on disease duration in sCJD population. We observed a significant longer disease duration in *PRNP* M129V heterozygous compared to *PRNP* homozygous (p<0.001) (M129M: 5.3±4.5 months, M129V: 13.1±7.7 months, V129V: 4.9±1.4 months). These results are in concordance with previous reports about the classification sCJD in several subtypes characterized by a combination of *PRNP* codon 129 status and the size of protease resistant PrP^Sc^ fragments and disease phenotype [31], [32]. On the other hand, we did not find any association with disease duration for either *APOE* ε2 allele or *APOE* ε4 allele carriers.

**Figure 1:**
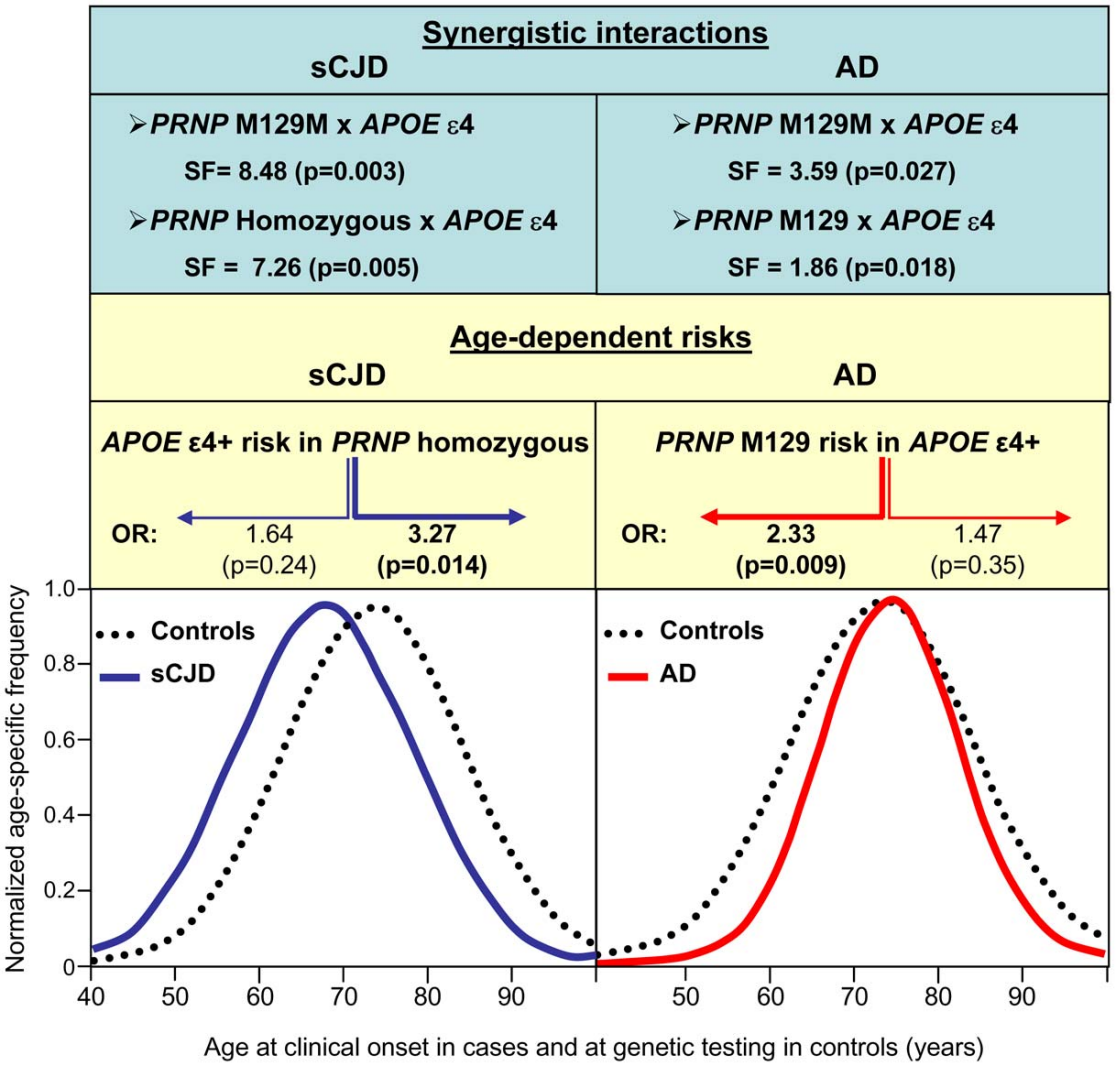
Synergistic age-dependent interaction between *APOE* and *PRNP* in both AD and sCJD. Top: Synergy factor values for the interaction *PRNP* codon 129×*APOE* ε4 in sCJD (left) and AD (right). Middle: Age-dependent risk. Left: sCJD associated with *APOE* ε4 allele in *PRNP* homozygous with onset before or after 71 years old. Right: AD associated with the *PRNP* M129 allele in *APOE* ε 4 carriers with onset before or after 74 years old. Bottom: Normalized age-specific frequency at clinical onset for sCJD (left) and AD (right) and at genetic testing in controls adjusted to a normal distribution curve.

We also study whether the synergistic effects observed between *APOE* ε4 and *PRNP* M129 was age dependent. For this purpose, we stratified cases and controls into two groups according to the median age for patients and control population pooled together. Analysis of AD risk related to the status of *PRNP* of the age stratified data indicated that the association to the *PRNP* M129 allele previously found in the *APOE* ε4 carriers stratum (see Table 3) was mainly determined by individuals with earlier onset (onset before 74 years old: OR = 2.33, 95% CI: 1.23–4.42, p = 0.009; onset at or after 74 years old: OR = 1.47, 95% CI: 0.66–3.25, p = 0.35) (see Figure 1, right).

Similarly, analysis of sCJD risk related to the *APOE* ε4 status of the age stratified data showed that the association for *APOE* ε4 previously found in the *PRNP* homozygous strata (see Table 6) was mainly driven by individuals with later onset (onset before 71 years old: OR = 1.64, 95% CI: 0.72–3.74, p = 0.24; onset at or after 71 years old: OR = 3.27, 95% CI: 1.27–8.43, p = 0.014) (see Figure 1, left).

## Discussion

A considerable number of studies have addressed association analysis of *PRNP* codon 129, a well-established genetic risk factor for sCJD, with AD; as well as the association of *APOE* ε4 allele, the major genetic risk factor for late onset AD, with sCJD. While the potential interaction between *APOE* and *PRNP* has been studied in AD populations, yielding controversial results [9], [11], [14], [16], [33], [34], few reports on the potential interaction between *APOE* and *PRNP* in sCJD have been published [24], [27]. The idea of undertaking a combined genetic analysis of sporadic AD and sCJD searching for interactions was motivated by reports on: i) the potential role of PrP in AD [35]–[41], and ii) because as brain amyloidoses, AD and sCJD, share several epidemiologic features such as an age at onset concordant with the clinical course duration, and vascular risk factors well established for AD and recently suggested for sCJD [3], [42], [43].

A part of our results mainly correspond to confirmatory data. Subjects bearing at least one *APOE* ε4 allele have 4.51-fold increased risk of developing AD in comparison with those that do not carry this allele (Table 2). Homozygosity at *PRNP* codon 129 increases risk of sCJD: 4.61-fold for M129M and 3.2-fold for V129V compared to M129V heterozygous (Table 5). Analysis of the risk attributable to *PRNP* in the overall AD population showed a statistically non-significant association (Table 2), of similar size and direction than the meta-analysis based on periodically updated data published at http://www.alzgene.org/ [44]. A similar analysis for the association of *APOE* in the overall sCJD population showed no association for the *APOE* ε4 allele (Table 5), nor for the *APOE* ε2 allele.

Hidden effects in overall analyses were unveiled by stratification according to their respective main risk genes (*APOE* ε4 allele status for AD, and *PRNP* genotypes for sCJD). We found statistically significant associations for the other gene in those strata at higher previous risk (*APOE* ε4 carriers for AD, and *PRNP* M129M homozygous for sCJD), suggesting an underlying interaction between *APOE* and *PRNP* as risk factors for both disorders (see Tables 3 and 6). The synergy factor analysis in AD and sCJD confirmed the interaction between these two genes as risk factors in both AD (SF = 3.59, p = 0.027), and sCJD (SF = 7.26, p = 0.005) (see Tables 4 and 7). Thus, subjects who carried a *PRNP* M129M genotype and at least one *APOE* ε4 allele for AD, or *PRNP* homozygous subjects that carried at least one *APOE* ε4 allele for sCJD have an increased risk that represent a deviation from a multiplicative model for the combined effects of these genetic factors.

It is understandable that a genetic interaction or epistasis given by statistical significant SF values does not necessarily correlate with a biological epistasis [45]. Provided there is a biological interaction, *PRNP* codon 129 appears to act a risk factor, at least in part, by different molecular mechanisms in AD and sCJD. While the risk of AD is associated with the *PRNP* M129 allele among *APOE* ε4 carriers, the increased risk of sCJD is associated with *PRNP* codon 129 homozygous (both M129M and V129V). Interestingly, we found that these interactions between *PRNP* and *APOE* were also age-dependent for both disorders, but in opposite directions (see Figure 1). While the increased risk of the *PRNP* M129 allele on *APOE* ε4 carriers was mainly associated with individuals with earlier AD onset; the *APOE* ε4 allele risk on *PRNP* codon 129 homozygous was mainly patent in the population with later sCJD onset. These results suggest that *APOE* and *PRNP* act as risk factors with different age-related timings, perhaps in correlation with differences in age-specific incidences for AD and sCJD. Thus, *PRNP* appears to be a genetic risk factor with an influence early during aging both in AD and sCJD, while *APOE* ε4 has a later expression. Several interrelated questions rise about the roles of *APOE* and *PRNP* interaction. First, if they determine the shape of the age-specific incidence curves shifting peaks in different directions; second, if they are related to disease course duration; third, if they are related to the vascular pathway and determine susceptibility pointing to same or different gene-environment interactions. Similarly, a recent report on rapidly progressive Alzheimer's disease has found that both *APOE* and *PRNP* may modulate the phenotypic expression of the disease [46].

Central to both neurodegenerative pathologies is an analogous proteolytic processing of a membrane protein (APP in AD, and cellular PrP in prion disorders) that generates unstable, aggregation prone, neurotoxic fragments that are pathognomonic for each disease (Aβ peptides and PrP^Sc^). The coexistence of AD and CJD pathology in a subgroup of sCJD patients [47], the colocalization of PrP^Sc^ and Aβ peptides in amyloid lesions both in sCJD and AD [48]–[52], and a synergist effect between AD and prion pathologies [53] have suggested common pathways involved in the generation or clearance of Aβ peptides and PrP^Sc^ [47]. In this sense, some appealing findings are starting to delineate shared regulatory mechanism that involves the amyloid beta peptides and their precursor protein (APP) and cellular PrP.

On one side, PrP regulates the levels of Aβ peptides by decreasing the cleavage of APP by BACE1 [35], [36]. On the other side, cellular PrP protein has been also identified as a high-affinity receptor for Aβ oligomers, the pathological species responsible for Alzheimer's disease, mediating their deleterious effects [37]–[41]. In order to reconcile the diverse roles of cellular PrP in Aβ metabolism, it has been proposed a feedback loop where, in normal brain, the levels of Aβ peptides regulates cellular PrP expression that in turn exerts an inhibitory effect on BACE1, decreasing Aβ levels. In AD, this feedback loop is disrupted by ever-increasing levels of Aβ oligomers that bind to cellular PrP shifting its interaction with BACE1, and therefore, preventing its regulation [35], [54].

Cerf and collaborators [55] observed that apoE4 strongly stabilizes Aβ oligomers, besides its reduced ability to clear these toxic forms of Aβ [56]–[59]. At genetic level, Combarros and collaborators [45] in a recent review about potential interactions between genes pairs in AD, reported that so far the only consistently replicated interaction was that between *BACE1* exon5 GG and *APOE4*. In this paper, we are reporting the interaction between *PRNP* M129M and *APOE4* in AD. Having in mind these facts, and based on the feed-back regulation discussed, we propose a potential mechanism for biological epistasis between the *APOE* ε4 allele and the *PRNP* M129 allele in AD. According to this model, we suggest that apoE4 preferential binding and stabilization of Aβ oligomers favour Aβ delivery to cellular PrP, instead of its targeting for cellular clearance; therefore disrupting the regulatory feed-back. Moreover, apoE has been shown to promote Dab1 phosphorylation that in turns regulates APP processing [60]. ApoE4 compared to apoE3 results in lower levels of phophorylated Dab1 and increased levels of Aβ peptides [58], [60]. In AD pathogenesis, Aβ oligomers may further sequester apoE, precluding regulation of APP processing mediated by Dab1, and promoting processing by BACE1 as above discussed.

The question whether the *PRNP* polymorphism affect the interaction of Aβ oligomers with cellular PrP was already posed by Kim and Tsai [61]. Interestingly, Parkin and collaborators [35] found an increase in Aβ_40_ peptide levels in brains of M129M mice compared to V129V, suggesting that cellular PrP M129 is less efficient inhibiting BACE1 activity. We propose that cellular PrP V129 could have a protective role in AD through a stronger inhibitory effect on BACE1 activity and weaker affinity for Aβ oligomers as a consequence of its particular structure.

As previously discussed, homozygosity at *PRNP* codon 129 is the main genetic driving force in sCJD disease [62] that correlates with a increased tendency to form toxic self-propagating PrP^Sc^ oligomers [63], [64]. A model where apoE4 stabilizes PrP oligomers, and impairs their clearance may also explain the synergistic association between *PRNP* and *APOE* in sCJD. Moreover, similar to apoE, PrP^Sc^ has been shown to affect to Dab1 phosphorylation and Aβ production [65], [66]. These data appear to explain the inverse correlation between PrP^Sc^ deposits and Abeta plaques in sCJD and animal models [35], [66]; and suggest new routes of convergence for AD and prion pathologies. Future studies are needed to elucidate the role of apoE4 in relation with cellular PrP and PrP^Sc^ oligomers for the development of the disease. However, analogously to AD, other effects of apoE-independent from PrP metabolism, such as cholesterol transport and synaptic plasticity and repair, and vascular implications may prove to be relevant for sCJD [67]–[70], [43].

In our opinion, this study has several features of relevance. We have investigated for the first time, *APOE* and *PRNP* genotypes simultaneously in the sporadic forms of AD and CJD compared to the same control population, with an age distribution intermediate between sCJD and AD cases. Potential bias by population stratification was minimized by selection of ethnically matched cases and controls from limited sources. Finally, the analysis of sCJD includes an important number of patients with definite diagnosis by anatomopathological study.

Unfortunately, the study has also limitations. Most important, the AD cases are selected solely on a clinical diagnosis basis; no anatomopathologically confirmed AD cases were included. Moreover, since our AD cases are not completely characterised by genetic analysis, some cases may correspond to genetic cases. However, given the low frequency of familial cases, and that all our AD cases are over 55 years with no aggregation of early onset cases in their family, it is unlikely that this fact may alter the results here discussed. Also, although the study populations are relative large, there are a small number of cases in some less frequent strata that may pose an increased risk of false positive results. Finally, only a limited sample of a Spanish population is included in the analysis, and therefore, perhaps the results are not suitable for other populations.

Discrepancies among results, including ours, can be related to ethnic differences or to the presence of additional factors influencing risk that are not included in the analyses [71]–[73]. Some authors have suggested that the lack of replication among different studies could be also related to epistasis or gene-gene interaction [45], [74], [75]. Of special relevance for this work, it is the study by Combarros and collaborators [12]; where opposite to our results, they did not find any association for the *PRNP* gene with the risk of developing AD, even after stratifying by *APOE* ε4 allele status in a Spanish population. Taking into account that even in our study the association effect is small; this discrepancy may be explained by the different geographic distribution of the studied populations that may play an important role in the interpretation of the results [16], [45], [76]. Thus, although both populations are of Spanish origin, Combarros' population is restricted to North of Spain, while ours have been recruited mainly from two different Spanish geographical areas (Eastern and Central areas). Perhaps most relevant, having in mind that we have found an age-dependent epistasis, the discrepancy between the two studies can be related to the different age distributions for the AD patients and controls included in these studies.

In summary, our data confirmed *APOE* ε4 and homozygosity at *PRNP* as major genetic risks for developing AD and sCJD, respectively. More interestingly, we found statistically significant age-dependent epistasis between these genes for both AD and sCJD, suggesting common pathways involved in the generation, clearance and neurotoxic signal transduction of Aβ peptides and PrP in AD and sCJD. Additional studies exploring larger populations are required to confirm our findings.

## Materials and Methods

### Patients and controls

Study population was composed of 474 AD patients diagnosed as “probable AD” according to NINCDS-ADRDA criteria [77] (patients with family history of dementia were not included), 175 sCJD patients and 335 control subjects with normal cognitive status. All subjects were Caucasians of Spanish origin (Eastern and Central areas). The study was approved by the Bioethics and Animal Welfare Committee (Comité de Bioética y Bienestar Animal) from the Instituto de Salud Carlos III, Madrid, Spain; and by the Ethical Research Committees (Comités de Ética en la Investigación) from the CIEN Foundation, and Universidad Autónoma de Madrid, Madrid, Spain. All samples for sCJD cases analyzed in this study were obtained from patients with suspected prion diseases, submitted for diagnostic purposes under the guidelines of the Spanish National Referral and Surveillance system. AD and control samples were obtained with the adequate understanding and written consent of subjects, family members or legal guardians, as appropriate. For this study, all samples were coded and personal information dissociated from the test results, according to local legislation at the time of analysis. All the data were analyzed anonymously, and all clinical investigations have been conducted according to the principles expressed in the Declaration of Helsinki.

### DNA analysis

Total DNA was isolated from peripheral blood or cerebral tissue following standard procedures. Genotyping of *APOE* polymorphisms (rs429358 and rs7412) was determined by Real-Time PCR [78] or with FRET probes [79] and the analysis of the polymorphism at codon 129 of the PRNP gene (rs1799990) was performed by DNA sequencing using specific primers [80].

### Statistical methods

Statistical analyses of nominal or categorical variables were performed by Fisher's exact text or Pearson's chi-square test. Quantitative variables (age at onset, disease duration) were analyzed by non-parametric statistical hypothesis contrast with Mann-Whitney U test. Additionally, logistic regression models controlled by age (as a linear variable) and gender were used to compare genotypic and allelic frequencies and to calculate association adjusted odds ratio (OR) and 95% confidence intervals (CIs). The Hardy-Weinberg test for genotype frequency distributions was performed on the observed genotype frequencies for population, with significance based on a standard observed-expected 2 with 1 df. The Mantel-Haenszel test was used to estimate the common odds ratio and to test whether the overall degree of association across different genotype strata was significant. Deviations from normality of quantitative variables were checked by the Kolmogorov-Smirnov statistic with Lilliefors' significance. The synergy factor (SF), confidence intervals and significance were calculated as previously described [45], [81]. All statistical analyses were performed with the GraphPad 4 or PASWStatistics 18 softwares.
